# A sweet taste receptor‐dependent mechanism of glucosensing in hypothalamic tanycytes

**DOI:** 10.1002/glia.23125

**Published:** 2017-02-16

**Authors:** Heather Benford, Matei Bolborea, Eric Pollatzek, Kristina Lossow, Irm Hermans‐Borgmeyer, Beihui Liu, Wolfgang Meyerhof, Sergey Kasparov, Nicholas Dale

**Affiliations:** ^1^ School of Life SciencesUniversity of WarwickCoventryUnited Kingdom; ^2^ Department of Molecular GeneticsGerman Institute of Human Nutrition Potsdam‐RehbrueckeArthur‐Scheunert‐Allee 114‐116Nuthetal14558Germany; ^3^ Transgenic Animal Unit, University Medical Center Hamburg‐EppendorfMartinistr. 52Hamburg20246Germany; ^4^ School of Physiology and PharmacologyUniversity of BristolUnited Kingdom

**Keywords:** energy balance, hypothalamus, tanycyte

## Abstract

Hypothalamic tanycytes are glial‐like glucosensitive cells that contact the cerebrospinal fluid of the third ventricle, and send processes into the hypothalamic nuclei that control food intake and body weight. The mechanism of tanycyte glucosensing remains undetermined. While tanycytes express the components associated with the glucosensing of the pancreatic β cell, they respond to nonmetabolisable glucose analogues via an ATP receptor‐dependent mechanism. Here, we show that tanycytes in rodents respond to non‐nutritive sweeteners known to be ligands of the sweet taste (Tas1r2/Tas1r3) receptor. The initial sweet tastant‐evoked response, which requires the presence of extracellular Ca^2+^, leads to release of ATP and a larger propagating Ca^2+^ response mediated by P2Y1 receptors. In *Tas1r2* null mice the proportion of glucose nonresponsive tanycytes was greatly increased in these mice, but a subset of tanycytes retained an undiminished sensitivity to glucose. Our data demonstrate that the sweet taste receptor mediates glucosensing in about 60% of glucosensitive tanycytes while the remaining 40% of glucosensitive tanycytes use some other, as yet unknown mechanism.

## 1 Introduction

Hypothalamic tanycytes are a specialized type of glial cell found lining the third ventricle (Bolborea and Dale, 2013; Rodriguez, Blazquez, Pastor, Pelaez, Pena, Peruzzo, & Amat, 2005). They have a cell body, which contacts the cerebral spinal fluid (CSF) and a single long process which projects into the hypothalamic parenchyma including the region of the main feeding centres—the arcuate nucleus and ventromedial hypothalamic nucleus (Bolborea and Dale, 2013; Rodriguez et al., 2005). Tanycytes are highly polarized cells that may be involved in transport of circulating hormones into the hypothalamus (Balland, Dam, Langlet, Caron, Steculorum, Messina, & Prévot, 2014; Langlet, Levin, Luquet, Mazzone, Messina, Dunn‐Meynell, … Dehouck, 2013a; Langlet, Mullier, Bouret, Prevot, & Dehouck, 2013b; Prevot, Langlet, & Dehouck, 2013). Owing to their unique position connecting the CSF to key hypothalamic regions, there has been growing interest as to the roles of hypothalamic tanycytes. Tanycytes, together with astrocytes, have a role in fatty acid storage and metabolism in the hypothalamus (Hofmann, Lamberz, Piotrowitz, Offermann, But, Scheller, … Kuerschner, 2017). Tanycytes may also help to regulate extracellular glutamate and lactate concentrations in the hypothalamic parenhcyma close to the third ventricle (Nilaweera, Herwig, Bolborea, Campbell, Mayer, Morgan, … Barrett, 2011). At least some tanycytes can act as diet‐responsive neural stem cells (Haan, Goodman, Najdi‐Samiei, Stratford, Rice, El Agha, … Hajihosseini, 2013; Lee, Bedont, Pak, Wang, Song, Miranda‐Angulo, … Blackshaw, 2012; Robins, Stewart, McNay, Taylor, Giachino, Goetz, … Placzek, 2013). A further potential role of tanycytes is that they sense glucose in the CSF.

Tanycytes in acute brain slices respond to glucose and nonmetabolisable glucose analogues when these are applied directly and selectively to the tanycyte cell body layer via puffing from a patch pipette (Frayling, Britton, & Dale, 2011). This puffing technique generated large repeatable increases in Ca^2+^ within the tanycytes, which spread in a wave like fashion between neighboring tanycytes (Frayling et al., 2011). The glucose‐evoked Ca^2+^ wave was found to depend on the release and extracellular diffusion of ATP acting via the P2Y1 receptor (Frayling et al., 2011). Further evidence for tanycytes as glucosensors comes from studies on primary cultures enriched in tanycytes, which respond to bath application of glucose also in an ATP‐receptor dependent manner (Orellana, Saez, Cortes‐Campos, Elizondo, Shoji, Contreras‐Duarte, … García, 2012). While these two studies show that tanycytes can respond to application of glucose, the sensing mechanism for glucose remains enigmatic.

Tanycytes have previously been shown to possess much of the same glucosensing machinery found in pancreatic β cells, possessing GLUT1 and GLUT2 glucose transporters, glucokinase and ATP‐sensitive K^+^ channels (Garcia, Carrasco, Godoy, Reinicke, Montecinos, Aguayo, … Nualart, 2001; Garcia, Millan, Balmaceda‐Aguilera, Castro, Pastor, Montecinos, …. Nualart, 2003; Millan, Martinez, Cortes‐Campos, Lizama, Yanez, Llanos, … García, 2010). Thus, a commonly accepted hypothesis is that tanycytes sense glucose through a mechanism similar to that used by pancreatic β‐cells (Bolborea and Dale, 2013; Dale, 2011; Orellana et al., 2012; Rodriguez et al., 2005). However, tanycytes also respond with Ca^2+^ waves to nonmetabolisable glucose analogues, 2‐deoxyglucose, and methyl glucopyranoside (Frayling et al., 2011). Another glucosensing mechanism must therefore be involved. Two alternative mechanisms have been proposed: reversal of the Na^+^/Ca^2+^ transporter; or activation of a G‐protein coupled receptor (Bolborea and Dale, 2013; Frayling et al., 2011).

According to the Na^+^/Ca^2+^ transporter hypothesis, movement of glucose into the cell via a Na^+^‐linked glucose transporter induces an influx of Na^+^, which in turn induces cell depolarization and may subsequently lead to the reversal of the Na^+^/Ca^2+^ exchanger moving Na^+^ out of, and Ca^2+^ into, the cell (Bolborea and Dale, 2013; Dale, 2011; Gonzalez, Jensen, Fugger, & Burdakov, 2008). The increase of Ca^2+^ inside the tanycytes may then, through an unknown mechanism, lead to release of ATP from the tanycyte, which may feed back onto the original activated cell leading to release of Ca^2+^ from intracellular stores, or stimulate neighboring cells thus propagating the Ca^2+^ wave (Bolborea and Dale, 2013; Frayling et al., 2011). While this is a possible explanation, the mechanism involved is rather complex.

Activation of G protein coupled receptors (GPCRs) provides a potentially simpler way of detecting extracellular glucose. The sweet taste receptors are a family of GPCRs well known for their ability to respond to sweet tasting compounds, including glucose. The sweet taste receptor is a heterodimer consisting of Tas1r2 and Tas1r3 receptor subunits (Li, Staszewski, Xu, Durick, Zoller, & Adler, 2002; Masuda et al. 2012; Nelson et al. 2001). It can bind glucose as well as a number of non‐nutritive sweeteners including sucralose and acesulfame K (Li et al., 2002; Masuda, Koizumi, Nakajima, Tanaka, Abe, Misaka, & Ishiguro, 2012). The sweet taste receptor has been well characterized in the tongue and expression of *Tas1r2* and *Tas1r3* has also been reported in the hypothalamus (Ren, Zhou, Terwilliger, Newton, & de Araujo, 2009). Owing to the expression of *Tas1r2* and *Tas1r3* in the hypothalamus and the affinity of the sweet taste receptor for glucose, this receptor represents an attractive candidate to mediate the glucosensitivity of tanycytes. In this article, we examine this hypothesis.

## 2 Materials and methods

### 2.1 Ethical approval

All experiments and procedures in this study were performed in strict accordance with the UK Animals (1986) Scientific Procedures Act and the project approved by the Animal Welfare and Ethical Review Board of the University of Warwick and under the authority of PPL 80‐2592, or with German national and institutional guidelines approved by the animal welfare committee of the Ministry of Environment, Health and Consumer Protection of the federal state of Brandenburg Germany (Permit Number 23‐2347‐A‐1‐2‐2010) and the animal welfare committee of the federal state of Hamburg (Hamburg, Germany; permit number G 21305/591‐00.33, No. 69/09).

### 2.2 Acute slice preparation

Male Sprague Dawley rats aged between 12 and 17 days and 6 weeks to 6 months old male B6 mice were humanely sacrificed by cervical dislocation in accordance with Schedule 1 of the Animals (Scientific Procedures) Act 1986. The brain was rapidly dissected and placed in ice‐cold artificial cerebrospinal fluid (aCSF 124 mM NaCl, 26 mM NaHCO_3_, 1.25 mM NaH_2_PO_4_, 3 mM KCl, 2 mM CaCl_2_, 1 mM MgSO_4_, 10 mM glucose saturated with 95% O_2_/5% CO_2_) with additional 10 mM MgCl_2_. The dissected brain was then placed under a microscope and the meninges covering the ventral surface of the brain were carefully removed using fine forceps. Coronal sections 300 µm thick were prepared using a vibrating microtome (Microm HM650). Each section was subsequently dissected along the midline separating the third ventricle and incubated in 35°C aCSF for 30–60 min to allow for recovery of adenine nucleotide levels (zur Nedden, Hawley, Pentland, Hardie, Doney, & Frenguelli, 2011). Slices were then transferred to 1 mM glucose aCSF (osmolarity maintained by addition of 9 mM sucrose) at room temperature for storage until required for imaging.

### 2.3 Viral constructs and injections

AdV‐pTSHR‐GCaMP3 was made by previously established methods (Duale, Kasparov, Paton, & Teschemacher, 2005) that involved cloning the 5′‐flanking region of rat thyrotropin receptor gene (Ikuyama, Niller, Shimura, Akamizu, & Kohn, 1992) into a dual promoter construct (Liu, Paton, & Kasparov, 2008). In strict accordance with the Animals (1986) Scientific Procedures Act, male C57BL/6 mice aged between 8 and 12 weeks old were maintained under deep anaesthesia via inhalation of Isofluran (Baxter). The level of anaesthesia was verified by testing of paw and tail withdrawal reflexes. The animals were placed in a stereotaxic frame (Kopf). A small hole was drilled in the skull to permit injection (via a 5 μL calibrated microcapillary tube, Sigma) of AdV‐pTSHR‐GcAMP3 into the lateral ventricle (2.5 – 5 × 10^9^ viral particles) at the stereotaxic coordinates: bregma 0 mm; midline – 0.72 mm; dorsal surface – 2.3 mm. After the procedure, a single injection of Metacam (Meloxicam) injectable (5 mg/ml; Boehringer Ingelheim) was given to the animal. The animals recovered for a week, and then acute slices were made, as described above.

### 2.4 Immunocytochemistry

After recording from slices, tissues were fixed in a solution of phosphate buffer 0.1 M plus 4% formaldehyde overnight (about 16 hr). The fixed slices were washed 3 times for 15 min with a phosphate buffer saline (PBS) solution. A blocking solution (PBS + 5% bovine serum albumin + 0.04% Triton‐X‐100) was applied for 1 hr and then directly followed by the primary antibodies as follows: GFP (for visualizing GCaMP3) 1:500 (ab6556) and Vimentin 1:500 (ab24525); GFP (1:500 ‐ ab6556) and NeuN 1:500 (Millipore, MAB377); GFP (1:500 ‐ ab6556) and Hexon protein 1:1000 (Pa1‐7201); GFP (1:500 ‐ ab6556) and glial fibrillary acidic protein (GFAP) 1:200 (AbCAM, ab4674). These primary antibodies have been well characterized as being specific by others: vimentin (Wang, Wang, Zhao, Ma, Rodriguez, Fariss, & Wong, 2014), hexon (Muck‐Hausl, Solanki, Zhang, Ruzsics, & Ehrhardt, 2015), GFAP (Nagao, Ogata, Sawada, & Gotoh, 2016), and NeuN (Radford, Moreno, Verity, Halliday, & Mallucci, 2015). The specificity of the GFP antibody was verified by demonstrating lack of staining of wild type tissue. Primary antibodies were incubated for 2 hr at room temperature. The slices were then washed three times in PBS before incubating with appropriate Alexa (Invitrogen) fluorescent conjugated secondary antibodies: donkey anti‐rabbit for GFP with either goat anti‐chicken (for Vimentin or GFAP), chicken anti‐goat (for Hexon), or goat anti‐mouse (for NeuN), all diluted a working concentration 1:1000. For all three antibody combinations, control slices were incubated only with secondaries. After three final washes in PBS and slices were mounted in VectaShield with DAPI (Vector Labs) on a microscope slide. Imaging was performed on a Leica SP5 confocal microscope, and a single optical section is shown, that documented the most intense staining for each of the markers.

### 2.5 Ca^2+^ imaging

To image Ca^2+^ changes in tanycytes, hypothalamic slices were incubated with the Ca^2+^ indicator Fura‐2 (12.5 µg/ml in 0.125% DMSO and 0.025% pluronic) for 90–120 min in 1.0 mM glucose aCSF. Loaded slices were transferred to a flow chamber containing circulating 1.0 mM glucose aCSF and imaged with an Olympus BX51 microscope using a 60× water immersion objective (NA 0.95). An Andor Ixon EM‐CCD camera was used to collect the images. A ratiometric image of Fura‐2 loading was achieved by illuminating at 340 and 380 nm with a xenon arc lamp (Cairn Research) and a monochromator (Optoscan, Cairn Research).

For live imaging of slices derived from mice expressing GCaMP3, the slices were mounted on a Scientifica Slicescope and observed via an Olympus 60x water immersion objective (NA 1.0). Illumination was provided via a 470 nm LED (OptoLED, Cairn Research) and a Hamamatsu ImageEM EM‐CCD camera was used to collect the images. Metafluor imaging software was used to control the illumination and camera in all experiments.

### 2.6 Application of sweet taste compounds

Glucose or non‐nutritive sweeteners were applied via puffing from a glass patch pipette (inner tip diameter 3–5 µm, applied pressure 60 kPa). For puffing experiments 290 mM glucose, 290 mM sucralose were dissolved into a solution of 10 mM HEPES pH 7.4 to give a solution with an osmolarity of 300 mOsm. 165 mM acesulfame K (AceK) was dissolved in 10 mM HEPES solution, pH 7.4, to give a solution with an osmolarity of 300 mOsm. A solution of 300 mM rebaudioside A (RebA) was diluted 1:1 with aCSF to give a final concentration of 150 mM in the patch pipette. The sweeteners were puffed directly onto the tanycytes in a series of 300–500 ms pulses spaced about 2 s apart. We previously established that this method gives a 30‐40 fold dilution of the pipette contents at the slice—as measured for glucose puffs by using a glucose biosensor to verify the applied concentration (Frayling et al., 2011). Thus the effective dose at the tanycytes for sucralose was ∼8 mM, and for RebA and AceK ∼4 mM. As a control, aCSF with 10 mM HEPES (0 mM glucose) was puffed onto the tanycytes. For every recording of a tastant response, the image focus and illumination at the 340 and 380 nm wavelengths was reoptimized and a baseline set of images acquired prior to application of the tastant.

### 2.7 Other pharmacological manipulations

ATP was applied to the slice through the bathing medium at a concentration of 10 µM. The P2Y1 receptor antagonist, MRS2500, was applied at 100 nM for ∼10 min prior to tastant stimulation. For experiments investigating the contribution of intracellular Ca^2+^, 20 µM cyclopiazonic acid (CPA) was added to the slice through the bathing medium for at least 20 min to ensure emptying of internal Ca^2+^ stores. To remove extracellular Ca^2+^ from the bathing media, the Ca^2+^ was replaced by 2 mM Mg^2+^ and 1 mM EGTA added to the aCSF. This zero Ca^2+^ aCSF was applied to the slice for 10 min prior to testing.

### 2.8 Data analysis

For Fura‐2 recordings, all analysis was performed on the ratio of the fluorescence at 340 and 380 nm (*F*_340_/*F*_380_). For GCaMP3, an initial fluorescent baseline signal was computed (*F*_0_) and subsequent fluorescent values (*F*) were normalized to this (*F*/*F*_0_). Analysis of imaging data was performed offline using ImageJ. Regions of interest (ROIs) were drawn around individual tanycytes (identified as described previously (Frayling et al., 2011) from the infrared and/or the fluorescence images at an excitation of 340 nm, examples shown in Figures 2, 3, and 7) and the average pixel intensity of each cell calculated. To calculate the magnitude of cell response induced by a stimulus (puffing of tastant), the average baseline value (consisting of at least 5 consecutive images) immediately prior to the stimulus was calculated. The maximum change in intensity was then subtracted from the baseline for each ROI that was seen to respond. A change from baseline of >0.01 was taken to be the minimum value of response when a response was clearly observed, thus changes below this value were excluded from analysis. Where no response was observed, (as in the case of inhibitors) the average values from a similar number of ROIs were taken. The mean value from each ROI averaged to produce one value for statistical analysis.

### 2.9 *Tas1r2* null mice

**Figure 1:**
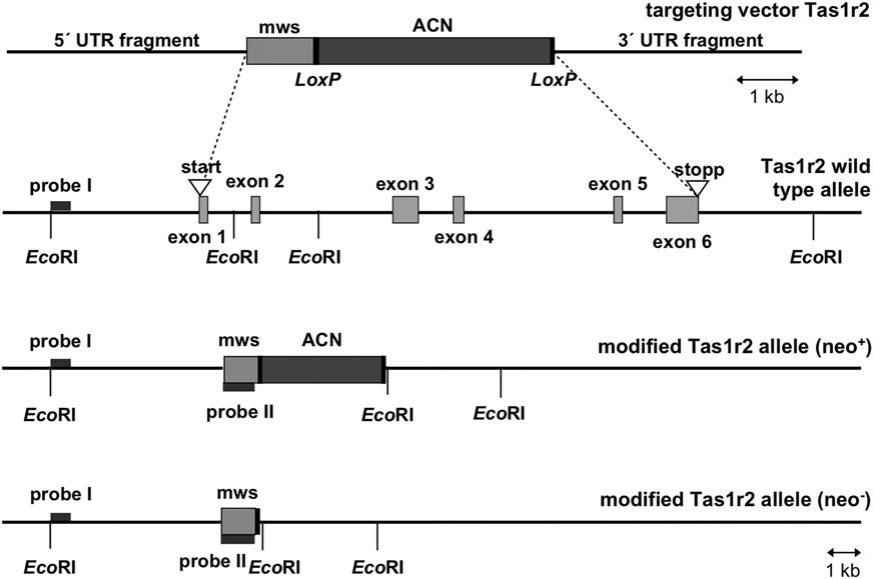
Generation of *Tas1r2* null mice. Schematic presentation showing the structure of *Tas1r2* gene and the strategy for generating knock‐out mice. From top to bottom, targeting construct, the *Tas1r2* wild type allele, the targeted *Tas1r2* allele before (neo^+^) and after (neo^−^) self‐induced deletion of the neomycin selection cassette (ACN) are shown. Light gray boxes represent coding sequences for either the *Tas1r2* gene or the inserted mouse *opsin 1 mws* gene. The inserted sequence was composed of the coding sequence for opsin mws and the ACN cassette. This cassette, flanked by *LoxP* sites, contained the testis‐specific angiotensin‐converting enzyme promoter to drive Cre‐recombinase expression, and a murine RNA polymerase II promoter to drive expression of the neomycin resistance gene

The strategy for generation of *Tas1r2* null mice is illustrated in Figure 1. The coding sequence of *Tas1r2* was exchanged by knocking in the coding sequence of mouse opsin 1 medium wavelength sensitive [mws, green cone pigment; GenBank: AF011389; (Sun, Macke, & Nathans, 1997; Yokoyama, 2000)]. Insertion was based on homologous recombination in 129/Sv stem cells. Therefore 5′ and 3′ fragments of the *Tas1r2* coding sequence were amplified by PCR using a bacterial artificial chromosome bMQ‐199P13 (Wellcome Trust Institute) derived from 129 mice strain as template, to amplify a 1.2 and 2.0 kb fragment, respectively. Homologous fragments were cloned into pKO‐V901‐diphtheria toxin A‐chain (DTA) plasmid (Lexicon Genetics), next to the coding sequence of the mouse *opsin mws* and a Cre‐recombinase‐ and neomycin resistance gene‐containing selection cassette (ACN) flanked by *Lox*P sites (Voigt, Hubner, Lossow, Hermans‐Borgmeyer, Boehm, & Meyerhof, 2012). The final targeting vector was electroporated into R1 embryonic stem cells (Nagy, Rossant, Nagy, Abramow‐Newerly, & Roder, 1993). The neomycin resistance gene in the ACN cassette and DTA gene of the plasmid backbone were used for positive and negative selection, respectively. 460 G418‐resistant colonies were selected; genomic DNA was digested with *EcoR*I and hybridized with a random‐primed alpha‐^32^P‐labeled probe on Southern Blot. To identify homologous recombinant stem cell clones an external probe (probe I) was used, amplified using oligonucleotides 5′‐TTCCCCGCCTGTCTGCTTTTCT‐3′ and 5′‐TCCATGTGGACCCCAGGCAAAT‐3′. Correctly targeted stem cell clone was injected into C57BL/6 blastocysts, and chimeric mice were bred with C57BL/6 animals. Genomic DNA of the offspring was genotyped by hybridization with an additional internal probe (probe II) on Southern Blot, validating the self‐induced deletion of the ACN selection cassette by the Cre‐loxP system. Internal probe was generated by using oligonucleotides 5′‐GGGACGTCGCCACCATGGCCCAAAGGCTTACAGGTGAACA‐3′ and 5′‐GGGAGATCTTTATGCAGGTGACACTGAAGAGACAGATGAGAC‐3′. Further genotyping based on PCR analysis with oligonucleotides 5′‐GACACCAAATGAATGGATGAGGCTG‐3′ and 5′‐TGAGGTGAGAGACGCTCTTCACGTT‐3′ for wild type and 5′‐GACACCAAATGAATGGATGAGGCTG‐3′ and 5′‐CCCAGAACGAAGTAGCCATAGATTTGG‐3′ for mutated allele, to amplify a 526 and 713 bp fragment, respectively. Heterozygous animals were interbred to produce homozygous offspring B6;129SvJ‐*Tas1r2* ^tm‐Opsin mws^. Genomic modification was further approved on RNA level by Reverse transcription polymerase chain reaction (RT‐PCR) in situ hybridization, performed as described previously for *Tas1r1* (Voigt et al., 2012). Oligonucleotide sequences for RT‐PCR were as follows: *Tas1r2* 5'‐AGAGTTGCCAGCCTGGGCAAAT‐3′ and 5′‐GAAAGTTGAGCACAGTGACCAG‐3′, *Opsin mws* 5′‐CCGGTTCATAAAGACATAGATAATGGGGT‐3′ and 5′‐AGACATCCTGTGGCCCAGACGTGTT‐3′, amplicon size 912 and 383 bp, respectively. Probe for *Tas1r2* was based on Allen Brain Atlas (Allen Institute for Brain Science), whereas probes for *opsin mws* contained the regions encoding either for exon 2 or exon 5, amplified by using oligonucleotides 5′‐GGGACGTCGCCACCATGGCCCAAAGGCTTACAGGTGAACA‐3′ and 5′‐TAAGGCCAGTACCTGCTCCAACCAAAGAT‐3′, next to 5′‐AGACATCCTGTGGCCCAGACGTGTT‐3′ and 5′‐GGGAGATCTTTATGCAGGTGACACTGAAGAGACAGATGAGAC‐3′. Tissue used for RT‐PCR and in situ hybridization based on animals on mixed 129/Sv and C57BL/6 genetic background, whereas mice used for analysis of tanycytes were backcrossed 10 generations on C57BL/6 (B6.129SvJ‐Tas1r2^tm‐Opsin mws^).

### 2.10 Comparison of *Tas1r2* null and wild type mice

Slices were cut from *Tas1r2* null mice or from wild type C57BL/6J mice, which is the closest wild type match to the genetic background of the knock out mice. A standardized protocol (size of puffer pipette, placement, number of stimuli) was used to test the responsiveness of slices from the two strains of mice. For analysis ROIs were drawn around every tanycyte in the field of view. This enabled quantification of the responses to glucose across all tanycytes (responsive and nonresponsive), only those tanycytes that responded, and the number of responsive and nonresponsive tanycytes in a slice. A total of 22 slices from 7 wild type mice and 24 slices from 8 *Tas1r2* null mice were used. The wild type and *Tas1r2* null mice were matched for age and body weight 25.5 ± 2.8 and 25.3 ± 2.9 g, respectively.

### 2.11 Statistical analysis and presentation

In the case of characterization of tanycytes responses to sweet tasting compounds and analysis of the signalling pathway one slice was regarded as an independent repetition. Thus responses from individual tanycytes in one slice were used to calculate a mean response for that slice. All graphs illustrate the median response with the error bars representing the lower and upper quartiles. Nonparametric statistics were used because many samples had wide variation in the magnitude of the control responses prior to experimental manipulations. To show that tanycytes respond to sucralose, AceK and RebA were compared with pooled data from control puffs. The Kruskal‐Wallis test was performed on the data followed by the Mann‐Whitney U test to identify whether responses to an individual sweetener were different from control or other sweeteners. For the matched inhibition studies Friedman 2‐way ANOVA was performed to compare the control, drug, and wash responses. The Wilcoxon Matched Pairs Signed Ranks test was then used for pairwise comparison of drug treatments if required. The False Discovery Rate procedure was used to check significance when multiple comparisons were made (Curran‐Everett, 2000).

To avoid pseudoreplication in the case of the comparison of wild type and *Tas1r2* null mice, the responses across all tanycytes and all slices from one animal was used to calculate the mean response for that animal, and the number of independent repetitions was thus the number of animals.

## 3 Results

To explore the potential role of the sweet taste receptor in tanycyte signalling, we investigated three sweet‐tasting compounds, which act as ligands for the sweet taste receptor: the artificial sweeteners sucralose and acesulfame K (AceK); and a natural sweetener derived from *Stevia rebaudiana*, RebA (Li et al., 2002; Masuda et al., 2012; Sclafani, Bahrani, Zukerman, & Ackroff, 2010). As our previous work had shown the importance of selective stimulation of the tanycyte cell bodies with glucose (Frayling et al., 2011), we used focal application of the sweet substances to the tanycyte somata via a puffer pipette. The approximate concentrations at the tanycyte cell bodies of sucralose, AceK, and RebA were 8, 4, and 4 mM, respectively (see Methods).

### 3.1 Tanycytes respond to non‐nutritive sweeteners

**Figure 2:**
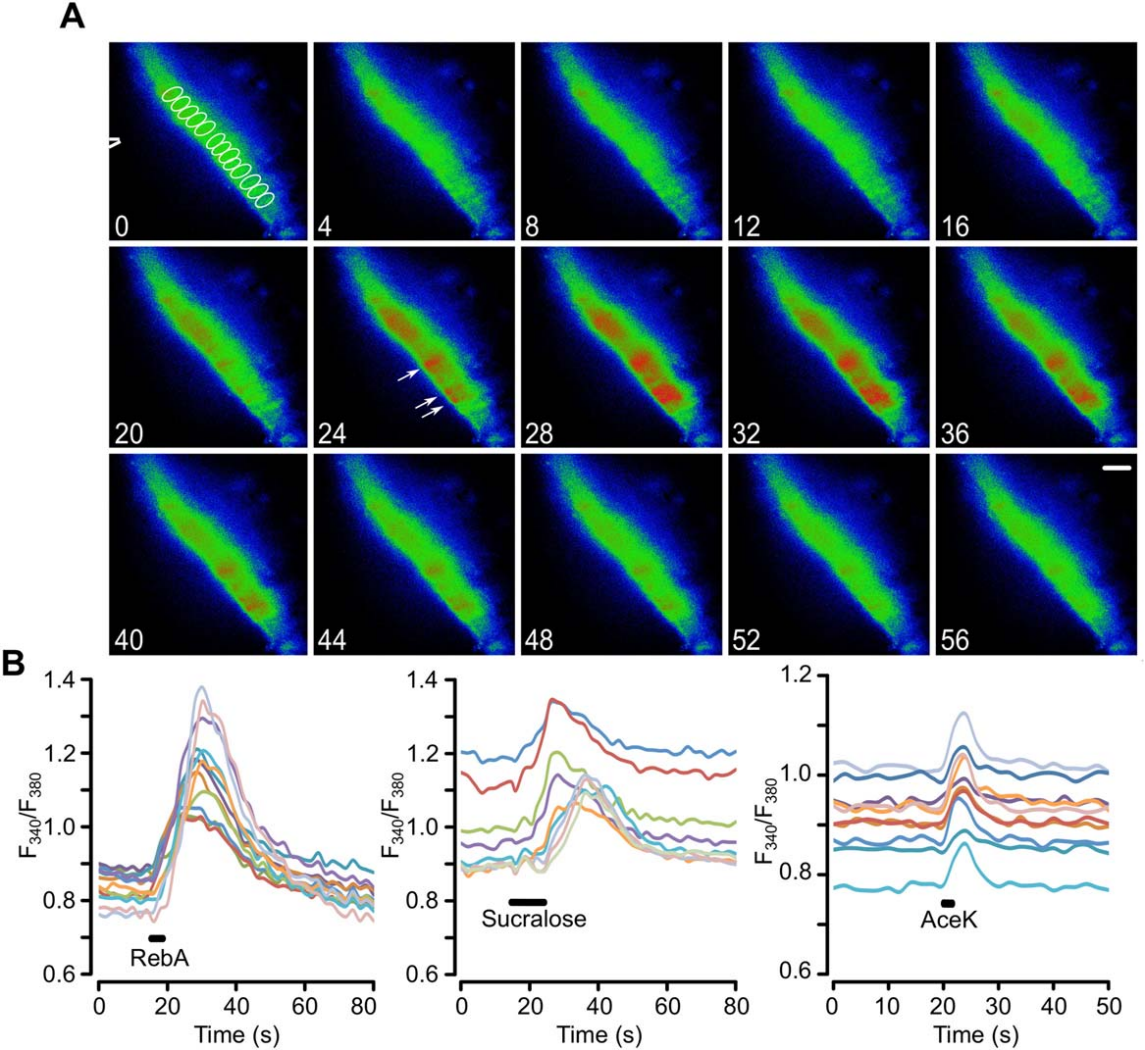
Tanycytes in hypothalamic slices from juvenile rats respond to non‐nutritive sweeteners. (a) Montage of pseudo color images showing the response of tanycytes to a puff of RebA, scale bar 20 µm. The tip of the puffer pipette is shown in the first image. The ROIs for drawn around individual tanycytes to measure their activation are indicated in the first image of the montage (in white). Numbers correspond to timings in (b). The white arrows indicate responses individual tanycyte cell bodies in which the Ca^2+^ elevation is clearly distinguishable during the response. (b) Quantification of the responses to RebA, sucralose, and AceK in ROIs drawn around individual tanycytes in the same slice for each graph. Each sweetener tested on a different slice. Only Ca^2+^ recordings from ROIs of responding tanycytes shown

Slices of rat brain were loaded with a Ca^2+^ reporter Fura 2‐AM. Tanycytes were identified by their morphology and location at the boundary of the third ventricle as previously documented (Frayling et al., 2011). The three sweet tasting compounds induced repeatable responses in hypothalamic tanycytes that were larger than those evoked by control puffs of artificial CSF (aCSF; mean change of *F*_340_/*F*_380_ for: control aCSF, 0.02 ± 0.006, *n* = 18; sucralose, 0.14 ± 0.03 *n* = 6, *p* = .0002; AceK, 0.05 ± 0.004 *n* = 6, *p* = .002; and RebA, 0.22 ± 0.04 *n* = 10, *p* = .0001; all comparisons to control aCSF puffs, Figure 2). Sucralose and RebA induced the characteristic Ca^2+^ waves first described for glucose and glucose analogues (Frayling et al., 2011). AceK also induced a response in the tanycyte layer; however, this response was rapid and short lived in comparison to the other sweet tasting compounds (Figure 2), which could often trigger responses that substantially outlasted the application of the sweet tastant.

**Figure 3:**
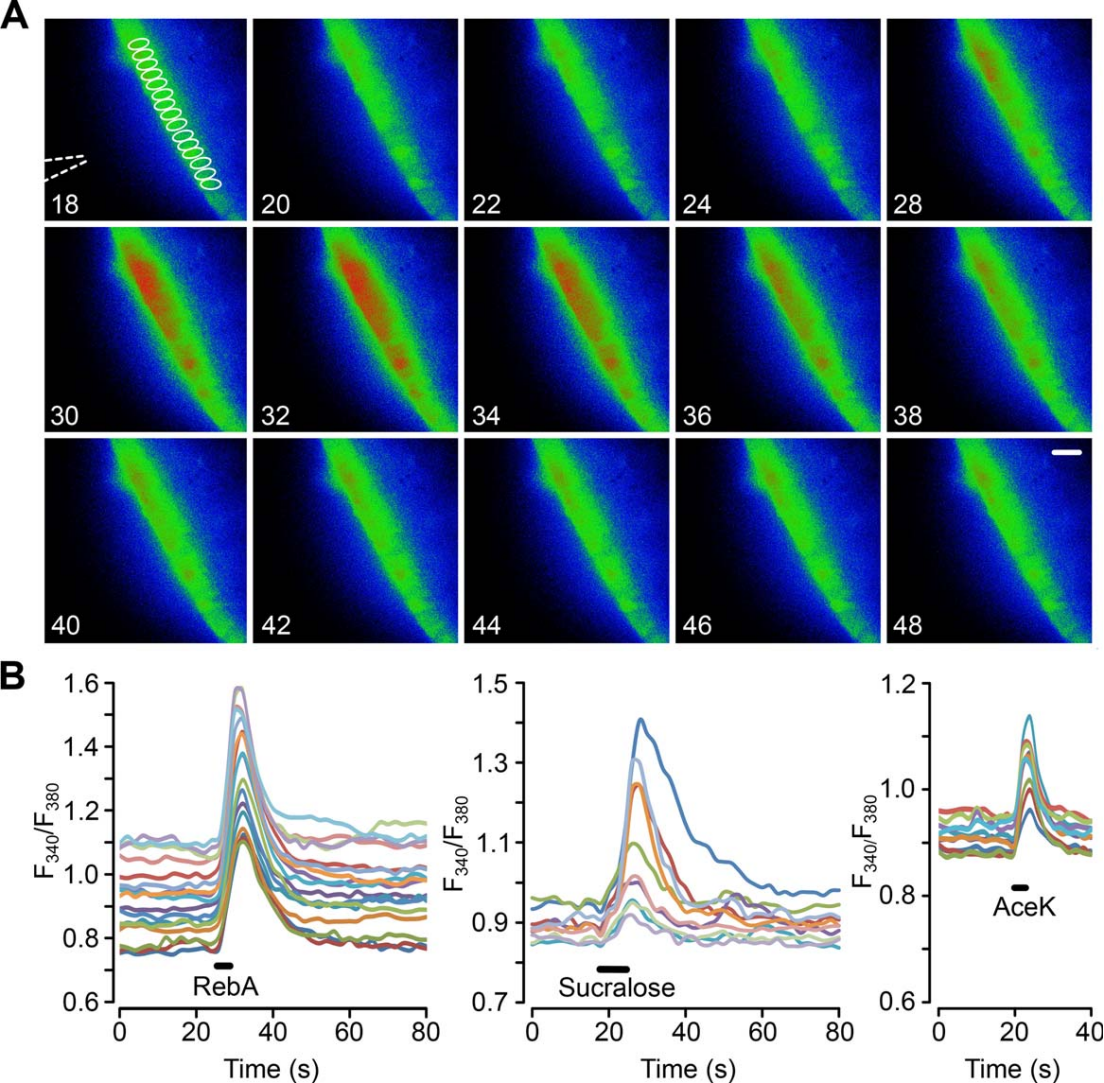
Tanycytes in hypothalamic slices from adult mice respond to non‐nutritive sweeteners. (a) Montage of pseudo color images showing the response of tanycytes to a puff of RebA, scale bar 20 µm. The tip of the puffer pipette is shown in the first image. The ROIs for drawn around individual tanycytes to measure their activation are indicated in the first image of the montage (in white). Numbers correspond to timings in (b). (b) Quantification of the responses to RebA, sucralose, and AceK in ROIs drawn around individual tanycytes in the same slice for each graph. Only Ca^2+^ traces from ROIs of responding tanycytes are shown. Each sweetener tested on a different slice

The responses to non‐nutritive sweeteners were not species or age specific. In slices derived from B6 wild type male mice aged between 6 weeks to 6 months (Figure 3)—sucralose, AceK, and RebA all induced responses in mouse tanycytes (mean change in *F*_340_/*F*_380_: control aCSF 0.02 ± 0.003 *n* = 13; sucralose 0.14 ± 0.002 *n* = 6; AceK 0.13 ± 0.03, *n* = 10; RebA 0.39 ± 0.13 *n* = 4). Interestingly the responses to AceK in tanycytes of mouse slices were much greater than those for rat. Tanycyte responses to non‐nutritive sweeteners are seen in both rats and mice and, at least in mice, are retained throughout juvenile and adult life. In a few experiments we cut parasagittal slices to image the apical surfaces of the tanycyte somata at the ventricular surface. This showed that the Ca^2+^ waves triggered by glucose and RebA spread radially in all directions (Supporting Information, Movie S1).

**Figure 4:**
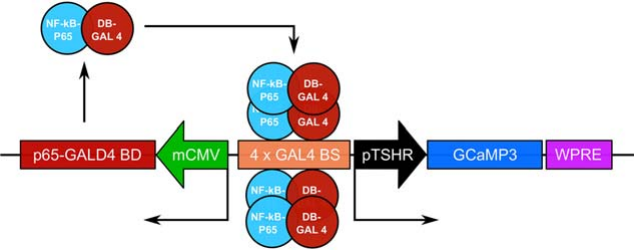
The AdV‐pTSHR‐GCaMP3 construct for targeting genetically encoded Ca^2+^ reporter to tanycytes [Color figure can be viewed at wileyonlinelibrary.com]

**Figure 5:**
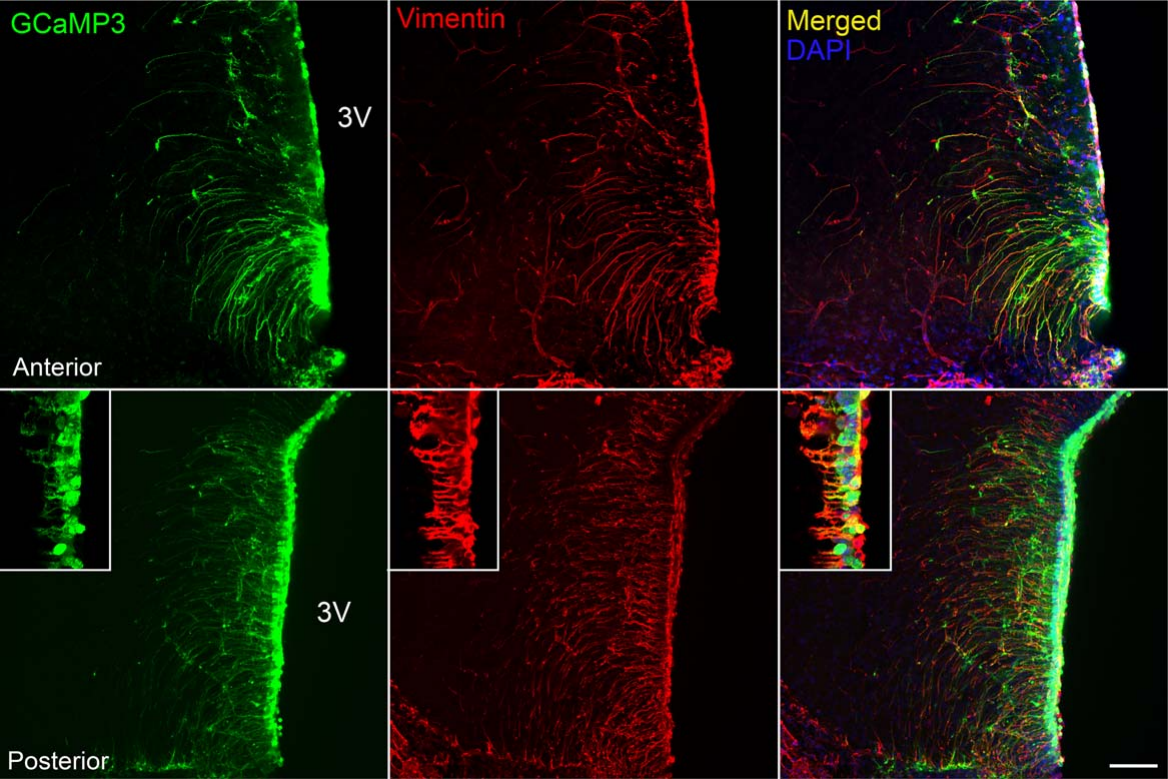
AdV‐pTSHR‐GCaMP3 specifically transduces tanycytes. Cells expressing GCaMP3 express vimentin, a marker of tanycytes, in cell body and soma, and have a morphology [soma lining the third ventricle (3V), and a single inwardly directed process] typical of tanycytes. The inset shows colocalization of vimentin with GCaMP3 in cell body and processes at higher magnification from different section. Scale bar 100 and 25 µm for inset

**Figure 6:**
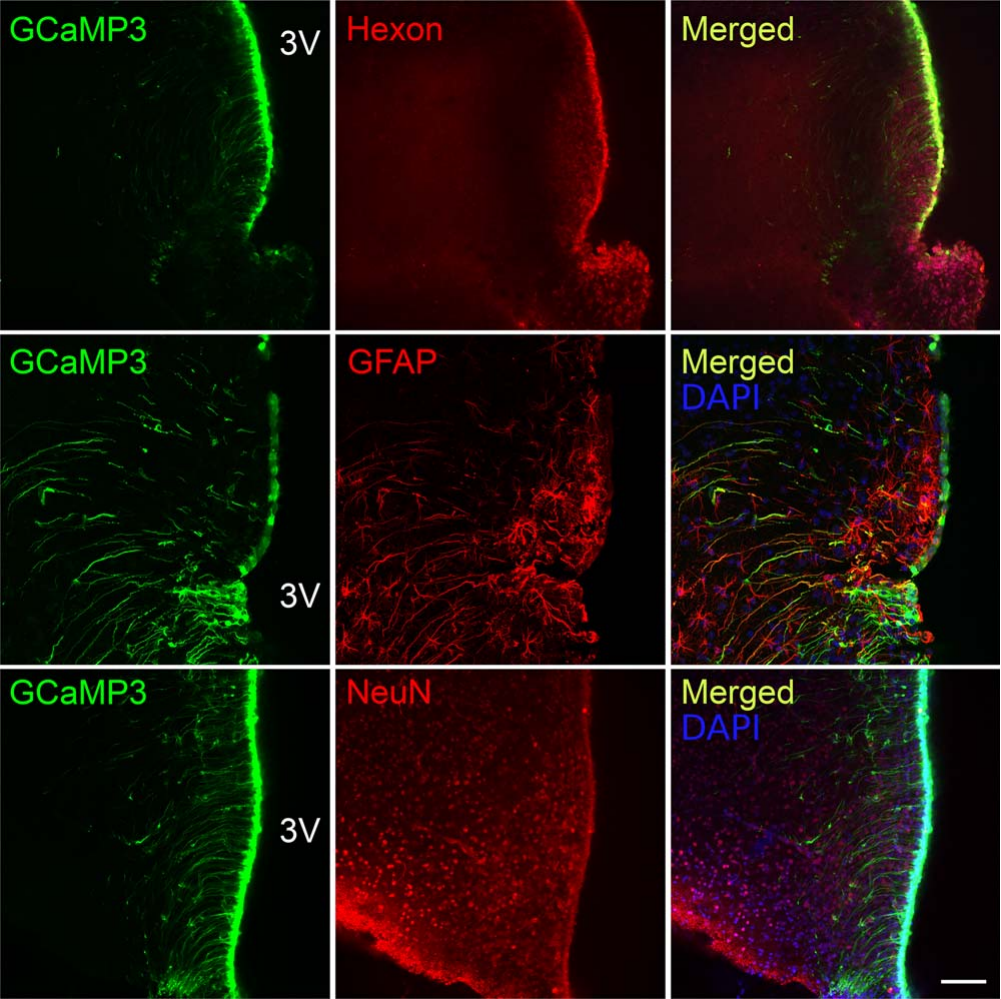
AdV‐pTSHR‐GCaMP3 does not transduce astrocytes or neurons. The cells that express GCaMP3 are a subset of the cells that express the hexon protein of the virus capsid, indicating selective promoter driven expression of GCaMP3 only in a subset of transduced cells. Astrocytes expressing GFAP do not express GCaMP3 even when close to ventricle wall. A subset of tanycytes also express GFAP. NeuN+ neurons in the brain parenchyma do not express GCaMP3. Scale bar 100 µm

**Figure 7:**
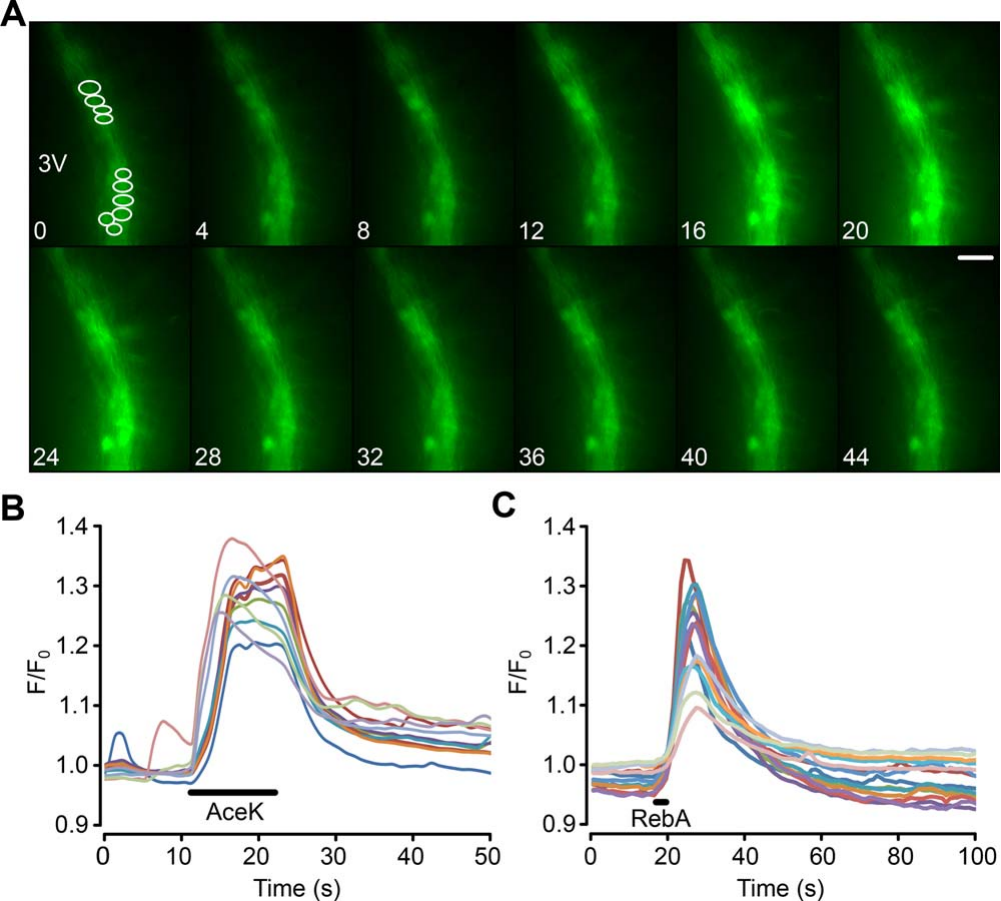
Genetically identified tanycytes in mice, expressing GCaMP3, respond to non‐nutritive sweeteners. (a) Montage showing tanycyte response to a puff of AceK. Fluorescence increases in both the cell bodies and the processes of the tanycytes. Numbers correspond to the time scale in (b). Scale bar 20 µm. 3V indicates third ventricle. ROIs shown in first image of montage. (b,c) Quantification of the responses to AceK and RebA in ROIs drawn around individual tanycytes. The analysis in (b) is from the same experiment as (a). (b,c) from different slices [Color figure can be viewed at wileyonlinelibrary.com]

In our studies, we have identified Fura 2‐loaded tanycytes by their characteristic morphology and position at the border of the third ventricle. To additionally verify that tanycytes do indeed respond to the non‐nutritive sweeteners, we targeted the expression of GCaMP3 (Tian, Hires, Mao, Huber, Chiappe, Chalasani, … Looger, 2009) specifically to tanycytes in B6 wild type male mice (8–12 weeks) with an adenoviral construct that used the TSH receptor promoter (Figure 4) to drive expression in these cells. The TSH receptor is selectively expressed in tanycytes in the hypothalamus (Hanon, Lincoln, Fustin, Dardente, Masson‐Pevet, Morgan, & Hazlerigg, 2008), and this ensures the tanycyte‐specific expression of GCaMP3 (Figures 5 and 6). Immunocytochemical staining of brain acute slices showed specific colocalization of GcAMP3 protein expression only in vimentin‐positive cells, lining the third ventricle (Figure 5). The GCaMP3 was not expressed in either GFAP‐positive cells in the brain parenchyma or NeuN‐positive cells in the brain parenchyma. Staining with the hexon antibody demonstrated the presence of viral particles in many parenchymal cells surrounding the ventricle. The specificity of the TSH receptor promoter is demonstrated because of the many cells that expressed the adenoviral hexon protein, only the tanycytes also expressed GcAMP3 (Figure 6). These genetically targeted tanycytes responded to both RebA and AceK with large increases in intracellular Ca^2+^ (Figure 7). The GCaMP3 expression in tanycytes also revealed that the tanycyte processes are capable of propagating Ca^2+^ signals in anterograde and retrograde directions (Supporting Information Movies S2 and S3).

### 3.2 Application of non‐nutritive sweeteners leads to activation of P2Y1 receptors

We previously demonstrated that tanycytes release ATP in response to glucose and that the extracellular diffusion of ATP propagates the Ca^2+^ wave along the tanycyte layer through activation of P2Y1 receptors (Frayling et al., 2011). We therefore used the selective P2Y1 receptor antagonist, MRS2500, to test whether the responses to sweet‐tasting compounds also involved ATP‐mediated signalling.

**Figure 8:**
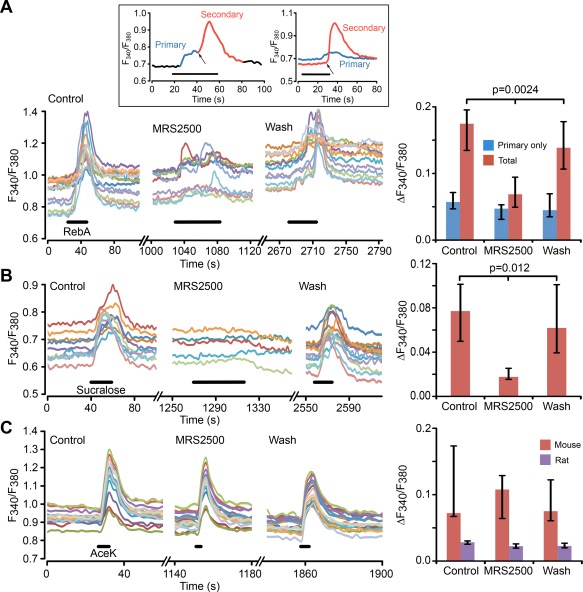
The secondary responses to non‐nutritive sweeteners depend upon activation of P2Y1 receptors. (a) 100 nM MRS2500 greatly reduces tanycyte responses to RebA. Left panel, ROI measurements from individual tanycytes in control MRS2500 and wash in the same slice. Right panel, histograms showing grouped data from 7 slices, which quantify the primary‐only and total responses to RebA. The primary responses are unaffected, but the total responses are reduced to about the same size as the primary only responses. Inset, shows examples of primary and secondary responses to non‐nutritive sweeteners. Inset, left: subdivision of the phases of a response to RebA (applied during black bar) into a primary (sweet receptor‐mediated, blue) and secondary response (ATP receptor‐mediated, red). Inset, right: an example where two tanycytes in the same slice individually exhibited only a primary and only a secondary response to RebA (applied during black bar). The arrows indicate onset of secondary responses. Note that in both cases the primary response has a slower onset than the secondary and that the secondary response can considerably outlast the period of application of tastant (black bar). (b) Left panel, MRS2500 (100 nM) almost completely blocks the responses to sucralose (ROI measurements from individual tanycytes in one slice). Right panel, summary histogram showing effect of MRS2500 on sucralose response from seven slices. (c) Left panel, MRS2500 (100 nM) has no effect of the tanycyte responses to AceK in mouse (ROI measurements from individual tanycytes in a single slice). Right panel, summary histogram, showing data from 6 slices for mouse and 3 slices for rat. All statistical comparisons made with Friedman 2 way ANOVA

MRS2500 at 100 nM greatly reduced responses to both sucralose and RebA (Figure 8a,b). In both cases a small remnant of the response to the sweeteners remained. Thus the responses activated by RebA and sucralose share a similar dependence on the P2Y1 receptor to the responses evoked in these cells by glucose (Frayling et al., 2011). If a GPCR were involved in generation of the responses to the sweeteners, we would expect to see a response resulting from the direct activation of the receptor that is independent of the extracellular actions of ATP. We noted that the responses to non‐nutritive sweeteners often appeared to be made up of two separate components: a slowly rising and lower amplitude initial response, which did not appear to propagate between cells; and a rapidly generated peak of much greater amplitude which propagated along the tanycyte layer and outlasted the period of stimulation by several seconds. We reasoned that the smaller initial response might be a change in intracellular Ca^2+^ due only to the activation of the hypothesized receptor, and termed this the primary response (Figure 8a, inset). We therefore examined responses to RebA that only consisted of the primary responses and defined these primary‐only responses as having a change in the *F*_340_/*F*_380_ ratio of <0.1. This definition of a primary response based on the criterion of amplitude <0.1 was then used in all subsequent analysis reported in the paper. MRS2500 had no effect on these primary‐only responses (Figure 8a), suggesting that they did indeed arise from the direct activation of a hypothesized GPCR. However, the responses to AceK in both rat and mouse were insensitive to MRS2500 (Figure 8c). This contrasts with the responses to RebA and sucralose and suggests that responses to AceK had no dependence on P2Y1 receptors.

### 3.3 The response to non‐nutritive sweeteners is depends on mobilization of intracellular Ca^2+^ and extracellular Ca^2+^

**Figure 9:**
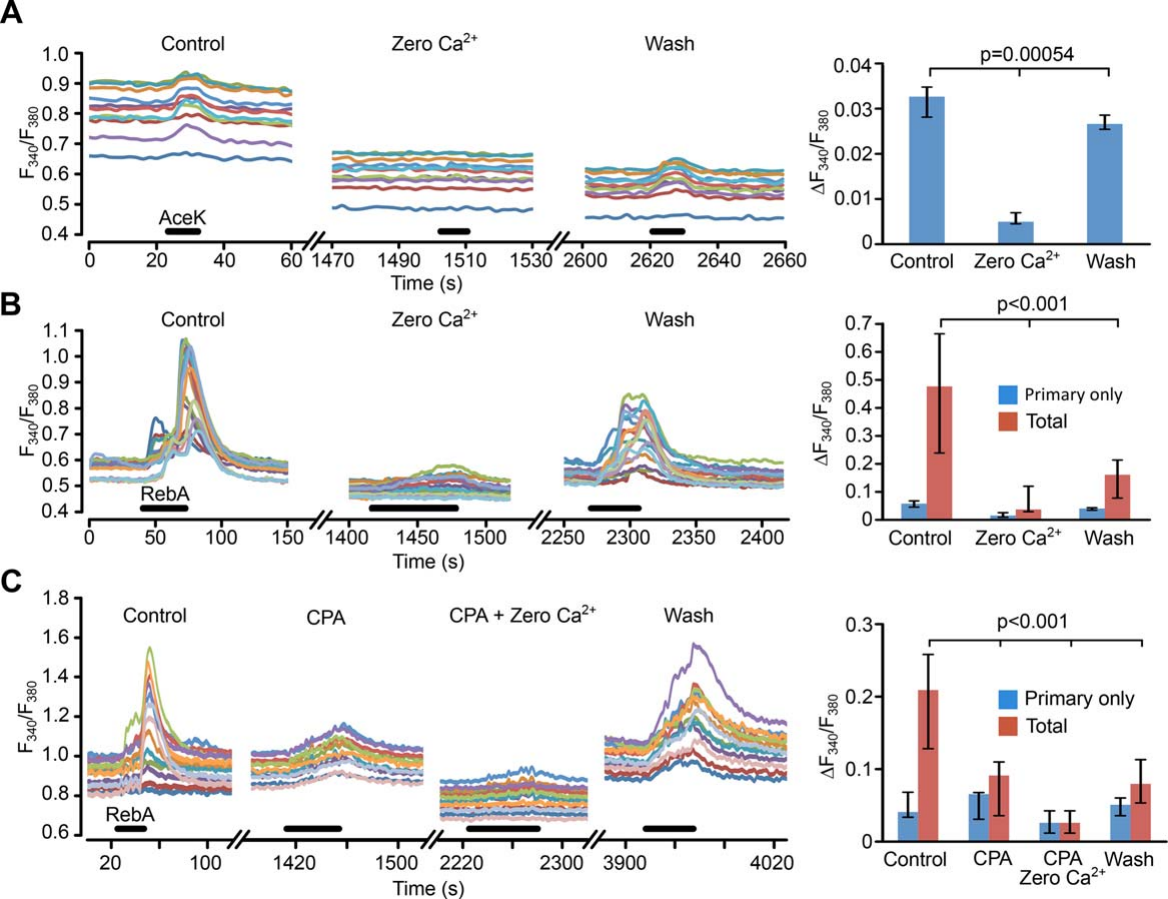
The responses to non‐nutritive sweeteners require extracellular Ca^2+^. (a, Left panel) Response to AceK in rat is almost completely abolished by removal of extracellular Ca^2+^ (ROI measurements from individual tanycytes in a single slice). (a, Right panel) Summary histogram showing data from 9 slices. (b, Left panel) Total response to RebA is greatly reduced by when extracellular Ca^2+^ is removed (ROI measurements from individual tanycytes in a single slice). (b, Right panel) Summary histogram showing data from 7 slices. The primary‐only responses (*n* = 4 slices) show the same trend, but this effect is not significant. (c, Left panel) Combination of emptying internal stores (CPA) and removal of extracellular Ca^2+^ gives a greater reduction of the total response to RebA than removal of extracellular CPA alone (ROI measurements from individual tanycytes in a single slice). (c, Right panel) Summary histogram from eight slices. All statistical comparisons made with Friedman 2 way ANOVA

We next investigated whether the sweet responses required extracellular Ca^2+^. Removal of extracellular Ca^2+^ abolished responses to AceK in rat (Figure 9a). The responses to RebA were also sensitive to the presence of extracellular Ca^2+^ and its removal greatly reduced the total response to RebA (Figure 9b). Removal of extracellular Ca^2+^ can depelete the Ca^2+^ levels in the internal stores. We therefore emptied intracellular Ca^2+^ stores by application of CPA, an inhibitor of the Ca^2+^‐ATPase. While CPA had no effect on the primary response, it greatly reduced the total response to about the same amplitude as the primary‐only responses (Figure 9c). This indicates that the responses do at least partly depend upon Ca^2+^ mobilization from intracellular stores. In the presence of CPA, the additional removal of extracellular Ca^2+^ caused a further reduction of both the primary‐only (*p* < .025 Wilcoxon Matched Pairs, *n* = 6 slices) and total responses (*p* < .025 Wilcoxon Matched Pairs, *n* = 7 slices). These data suggest that the responses evoked by activation by AceK and RebA most likely involve mobilization of Ca^2+^ from intracellular stores and an influx of extracellular Ca^2+^, possibly through G protein‐mediated activation of a nonselective cation channel.

### 3.4 *Tas1r2* and *Tas1r3* mRNA is present in the hypothalamus and the tanycyte layer

**Figure 10:**
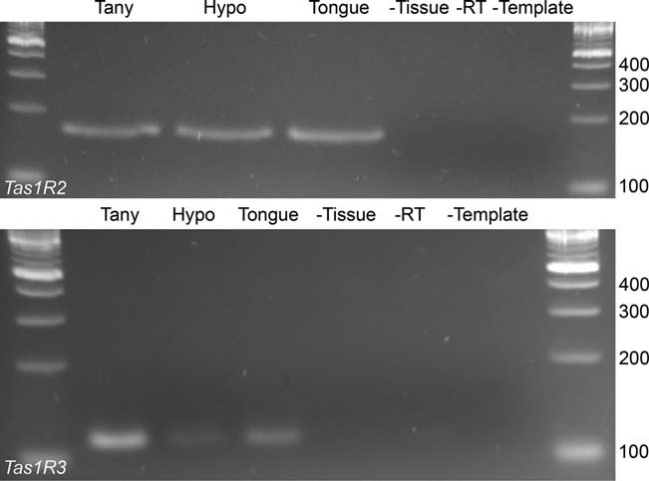
RT‐PCR data for *Tas1r2* and *Tas1r3* expression in the tanycyte layer and the hypothalamus. Primers for *Tas1r2* and *Tas1r3* gave PCR products of the expected size. The identities of the amplicons were confirmed by sequencing

The physiological responses in tanycytes evoked by the sweet‐tasting compounds suggest that the sweet taste receptors are present in tanycytes. We therefore performed RT‐PCR to test for the expression of *Tas1r2* and *Tas1r3*. With the aid of a microscope, we dissected tissue immediately adjacent to the boundary of the third ventricle to obtain samples highly enriched in tanycytes and compared this to the more lateral regions of the hypothalamus and the tongue. We found *Tas1r2* and *Tas1r3* expression in the tanycyte layer, the wider hypothalamus and, as expected, in the tongue (Figure 10). While this method does not give precise cellular localization it supports the hypothesis that tanycytes express the sweet taste receptor.

### 3.5 Characterization of *Tas1r2* null mice

**Figure 11:**
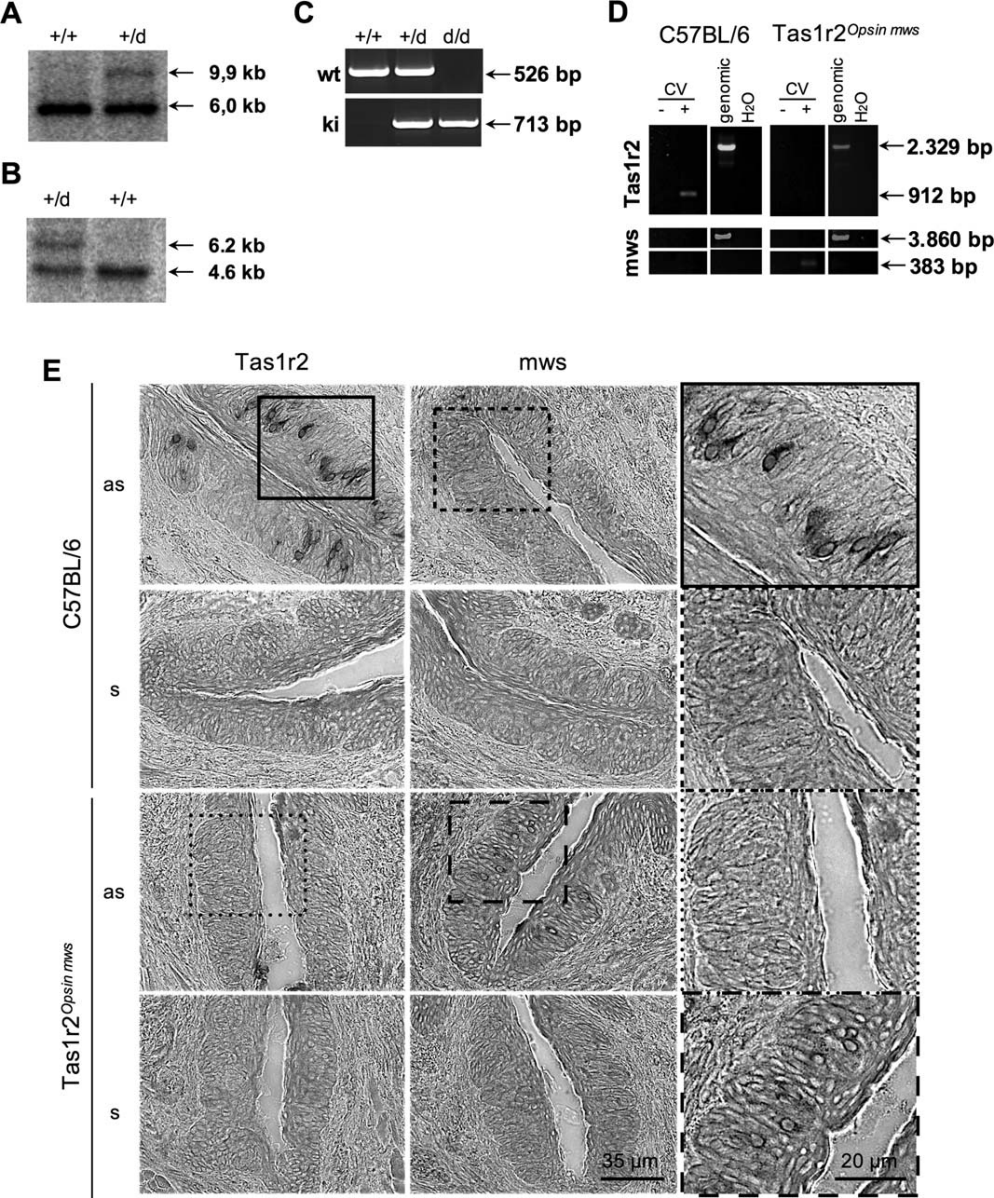
Characterization of *Tas1r2* null mice. (a,b) Genomic Southern Blot analysis of *Tas1r2* ^+/+^ and *Tas1r2* ^+/d^ mice. *EcoR*I‐digested genomic DNAs extracted from wild type or heterozygous animals were subjected to Southern Blot analysis with 5′‐flanking probe I (a) that distinguishes wild type and knock in alleles for *Tas1r2* or internal probe II (b) verifying the self‐excision of the ACN cassette by Cre‐recombinase. Probes I and II indicated in Figure 1. (c) Genotype analysis of *Tas1r2* mice. PCR products identifying genotype of *Tas1r2* mouse line based on specific oligonucleotides. (d) RNA isolated from lingual papillae (here vallate papillae, CV) were subjected to cDNA synthesis in or without presence of reverse transcriptase. PCR product specific for *Tas1r2* was only detected in wild type animals, whereas opsin mws was exclusively detected in *Tas1r2* null mice in gustatory tissue. (e) In situ hybridization analysis of tissue sections of *Tas1r2* animals. Tissue sections of vallate papillae of C57BL/6 wild type and homozygous *Tas1r2* null mice were hybridized with digoxigenin‐labelled riboprobes, recognizing *Tas1r2* and *opsin mws*. Tissue sections of C57BL/6 mice showed robust labeling when hybridized with *Tas1r2* antisense riboprobe (as). However, no signal was detected after hybridization with *opsin mws* as probe. In comparison to that, in tissue sections of homozygous *Tas1r2* null mice no labelling was detected when hybridized with *Tas1r2* as riboprobes, whereas hybridization with opsin mws as riboprobes resulted in the labelling of a comparable number of cells, indicating the successful knock in of mws and knock out of *Tas1r2*. Tissue sections hybridized with corresponding sense riboprobes did not show any labeling

Given the evidence for possible involvement of the sweet taste receptor, we next examined the ability of tanycytes to sense glucose in *Tas1r2* null mice. These animals carry a recombinant *Tas1r2* allele in which the *Tas1r2* open reading frame was replaced by that of opsin mws. Southern Blot analysis revealed the identification of one positively targeted stem cell clone (Figure 11a). This clone was injected into C57BL/6 blastocysts and chimeric mice were bred with C57BL/6 animals. Resulting offspring showed desired modification, indicating successful germline transmission and removal of the neomycin resistance cassette (Figure 11b). Heterozygous animals were interbred to produce homozygous offspring, genotyped by PCR (Figure 11c).

Initial characterization of the animals by RT‐PCR analysis revealed the absence of RNA for *Tas1r2* in lingual tissue of *Tas1r2* null mice, whereas Tas1r2 RNA was detectable in wild type animals. In contrast, RNA for *opsin mws* was undetectable on the tongue of C57BL/6 mice, but was found in *Tas1r2* null animals (Figure 11d). Moreover, we were able to visualise *Tas1r2* RNA but not *opsin mws* RNA in a subset of taste cells in vallate papillae of C57BL/6 mice by in situ hybridization (Figure 11e). In the null mice *Tas1r2* mRNA was absent, whereas a comparable number of *opsin mws* RNA‐positive cells were seen (Figure 11e). Together these results clearly indicate the absence of the Tas1r2 in the null mice.

### 3.6 Comparison of glucosensitivity of tanycytes from *Tas1r2* null and wild type mice

**Figure 12:**
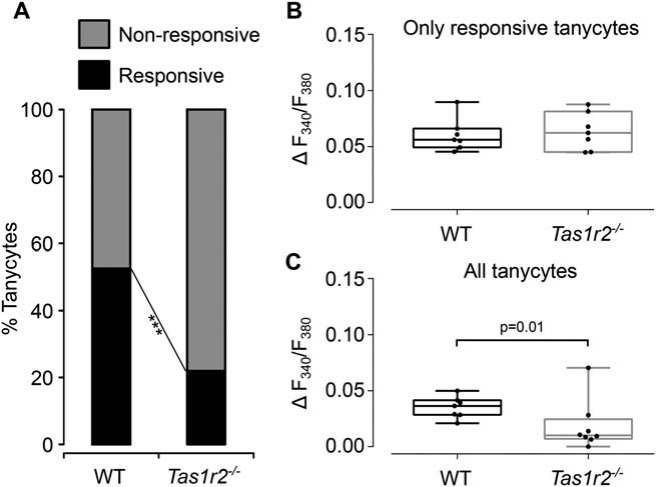
The sweet taste receptor mediates glucosensing in hypothalamic tanycytes. (a) Proportion of glucosensitive tanycytes is greatly reduced in *Tas1r2* null mice compared with wild type mice. (b) The magnitude of glucose responses in tanycytes that still respond in the *Tas1r2* null mice are the same as those in wild type mice. (c) When glucosensitivity of all tanycytes is computed (i.e., including those that do not respond as well as those that do) the overall response magnitude is greatly reduced. *** *p* < .0001 *χ* ^2^ test; in (b and c) each dot represents the mean tanycyte response amplitude to glucose for a single mouse. Box and whisker plots show the median, upper, and lower quartiles and range

In slices from wild type mice 292/555 tanycytes responded to puffs of glucose. In slices from the *Tas1r2* null mice only 153/696 tanycytes responded to glucose. These proportions were significantly different between the two strains of mice (Figure 12a, *p* < .0001, *χ* ^2^ test). When the magnitude of only the cells that responded to glucose was examined the mean change in *F*_340_/*F*_380_ was 0.06 ± 0.015 (*n* = 7) in wild type and 0.064 ± 0.017 (*n* = 8) in the *Tas1r2* null mice (Figure 12b). Thus in the knock out mice those tanycytes that remained glucosensitve, responded to glucose by the same magnitude as those in the wild type mice. However, when the response was calculated over all tanycytes in a field of view including both responsive and nonresponsive cells, then the mean change in *F*_340_/*F*_380_ was 0.035 ± 0.0097 (*n* = 7) in wild type and 0.0184 ± 0.0224 (*n* = 8) in the *Tas1r2* null mice (Figure 12c). Clearly in the knock out mice the overall mean response to glucose in the tanycytes population is greatly reduced compared with the wild type, reflecting the greater proportion of nonresponsive tanycytes in the knock out mice.

We conclude from these data that there are at least two independent mechanisms of glucose sensitivity in tanycytes: one that requires the sweet taste receptor, and a second that is independent of this receptor. Interestingly our data suggests that tanycytes detect glucose by either one of these mechanisms but not both, as the sensitivity to glucose of responsive tanycytes remains unchanged in the knock out mice.

## 4 Discussion

### 4.1 Glucosensing mechanisms in tanycytes

Our evidence shows that a substantial majority of tanycytes sense glucose via the sweet taste receptor. In addition to glucose and non‐metabolizable analogues of glucose, tanycytes also respond to three different ligands of the sweet taste receptor: sucralose, AceK, and RebA. We have isolated mRNA transcripts for the Tas1r2 and Tas1r3 receptor subunits from explants of the tanycyte layer, as well as the wider hypothalamic region. Crucially, in *Tas1r2* null mice the proportion of glucose‐insensitive tanycytes has increased. In the wild type mice, ∼53% of tanycytes responded to glucose puffs, this percentage was reduced by more than half in the *Tas1r2* null mice to ∼22%. These proportions suggest that 58% of glucosensitive tanycytes utilize the sweet taste receptor, and 42% some other mechanism to sense glucose.

Strangely, our initial assessment of whether tanycytes could still respond to the non‐nutritive sweeteners in *Tas1r2* null mice suggested that, unlike responses to glucose, the responses to RebA and sucralose were unaffected by this gene deletion. The non‐nutritive sweeteners RebA, AceK, and sucralose also have agonist activity at bitter taste receptors (Hellfritsch, Brockhoff, Stahler, Meyerhof, & Hofmann, 2012; Schiffman, Booth, Losee, Pecore, & Warwick, 1995). We have recently found that tanycytes respond to bitter tasting compounds and that these responses cannot be appreciably blocked by MRS2500 alone (E. Pollatzek, B. Webber, G. Lazutkaite, and N. Dale, unpublished observations). On the face of it this would suggest that the evidence that led us to evaluate the role of the sweet taste receptor (the responses evoked by non‐nutritive sweeteners) did so for the wrong reasons. In wild type tissue AceK responses were not blocked by MRS2500, indicating that this pathway differs from that of glucose, which is sensitive to this compound and may be via bitter taste receptors. However, the responses in wild type rats to RebA and sucralose were blocked by MRS2500, which also substantially blocked the Ca^2+^‐wave evoked by glucose (Frayling et al., 2011), suggesting a similarity of signalling pathway between RebA, sucralose, and glucose in the wild type. The apparent lack of effect of *Tas1r2* deletion on the responses to RebA and sucralose could therefore be due to an alternative agonist action on the bitter taste receptors in the knock out, or because these compounds act at some as yet uncharacterized receptor for sweet compounds (Simon, Parlee, Learman, Mori, Scheller, Cawthorn, … MacDougald, 2013). Given that these compounds are unlikely to enter the brain in any appreciable quantities following ingestion into the gut (Roberts and Renwick, 2008; Roberts, Renwick, Sims, & Snodin, 2000; Wheeler, Boileau, Winkler, Compton, Prakash, Jiang, & Mandarino, 2008), they are not agonists of physiological significance and we have concentrated only on the importance of the Tas1r2 receptor for responses to the physiologically relevant agonist glucose.

We have considerably extended our understanding of how tanycytes sense glucose, and introduced a new signalling paradigm for glucosensing in the brain. It remains to be seen if the signalling mechanism in tanycytes resembles that of taste buds. In the transduction pathway of the taste receptor cells, sweeteners acting via the sweet taste receptor activate phospholipase C‐beta2, inositol trisphosphate, inositol trisphosphate receptor type 3 and release calcium. In taste receptor cells, the Ca^2+^‐sensitive monovalent cation channel Trpm5 is required for depolarization of the cell and Trpm5 null mice are largely “taste blind” (Damak, Rong, Yasumatsu, Kokrashvili, Perez, Shigemura, … Margolskee, 2006; Kaske, Krasteva, Konig, Kummer, Hofmann, Gudermann, & Chubanov, 2007; Talavera, Yasumatsu, Yoshida, Margolskee, Voets, Ninomiya, & Nilius, 2008; Zhang, Hoon, Chandrashekar, Mueller, Cook, Wu, … Ryba, 2003). Eventually, ATP is released from sweet taste receptor cells to activate the gustatory nerves (Chaudhari and Roper 2010). We have not explored whether any of these signalling molecules might also be involved in tanycyte responsiveness to sweeteners, however, signal transduction in these cells also eventually leads to the release of ATP.

Previous authors have suggested that tanycytes sense glucose in a manner analogous to the pancreatic β cell, because the components of that mechanism (the relevant glucose transporters, glucokinase, the K^+^‐ATP channels, and SUR subunits) are present in tanycytes (Bolborea and Dale, 2013; Rodriguez et al., 2005). Our findings demonstrate that a different signalling pathway is used in the majority of glucosensitive tanycytes, and provide the first demonstration of signalling by sweet taste receptors in the brain. Nevertheless, it remains plausible that a mechanism dependent on glucokinase, the K^+^‐ATP channels, and SUR subunits accounts for glucosensitivity in the remaining glucosensitive tanycytes (Orellana et al., 2012). However, these mechanisms must be mutually exclusive as in the *Tas1r2* null mice tanycytes are either fully glucosensitive (a minority) or glucose insensitive (the majority). If both mechanisms were to coexist in the same tanycytes then a partial loss of sensitivity would be apparent in all glucosensitive tanycytes. It is interesting to note that this may be a further similarity between tanycytes and taste cells. Taste cells also possess two distinct sugar sensing mechanisms: the Tas1r‐dependent pathway; plus a second pathway that is independent of Tas1r but instead involves glycosidases, GLUTs, SGLT1, and an ATP‐gated K^+^ channel and is thus reminiscent of the glucose sensing mechanism in the pancreas (Sukumaran, Yee, Iwata, Kotha, Quezada‐Calvillo, Nichols, … Margolskee, 2016; Yee, Sukumaran, Kotha, Gilbertson, & Margolskee, 2011).

### 4.2 Roles of sweet taste receptors

Several reports have documented the expression of Tas1r receptor family subunits in extraoral tissues such as the gut, pancreas (Kyriazis, Soundarapandian, & Tyrberg, 2012) and in adipocytes (Laffitte, Neiers, & Briand, 2014). Knock out of the *Tas1r2* gene affects the regulation of insulin secretions from pancreatic β cells (Kyriazis, Soundarapandian, & Tyrberg, 2012). Deletion of the *Tas1r2* gene alters how mice adapt to a high fat low carbohydrate diet (Smith, Hussain, Karimian Azari, Steiner, Ayala, Pratley, & Kyriazis, 2016). Although these studies have considered the roles of these receptors in peripheral tissues, our discovery that tanycytes detect glucose via the sweet taste receptors suggests that contribution of a central mechanism may need to be examined. The Tas1r2/Tas1r3 receptor has been described in adipocytes, however, the actions of artificial sweeteners on these cells appears to occur via a pathway independent of these receptors (Simon et al., 2013) and thus has similarity to the results we report here. This raises the prospect that additional receptors for sweet tasting substances exist and may provide an additional hypothesis as to why some tanycytes retain sensitivity to glucose when the Tas1r2 subunit is deleted.

### 4.3 Glucosensing cells in CNS

A variety of cells sensitive to glucose have been described in the CNS. In the arcuate nucleus and ventromedial hypothalamus, neurons excited by glucose use a mechanism dependent on glucokinase and ATP‐sensitive K^+^ channels (Dunn‐Meynell, Routh, Kang, Gaspers, & Levin, 2002; Hussain, Richardson, Ma, Holton, De Backer, Buckley, … Gardiner, 2015; Levin, 2002; Marty, Dallaporta, & Thorens, 2007; Song and Routh, 2005; Wang, Liu, Hentges, Dunn‐Meynell, Levin, Wang, & Routh, 2004). Neurons in the lateral hypothalamus are also excited by glucose, although this may depend on both an ATP sensitive K^+^ channel and an additional mechanism involving the sodium‐linked glucose transporter (Gonzalez, Reimann, & Burdakov, 2009; Williams, Alexopoulos, Jensen, Fugger, & Burdakov, 2008). In addition to neurons, astrocytes can also respond to glucose (Leloup, Allard, Carneiro, Fioramonti, Collins, & Penicaud, 2016) and, in the ventrolateral preoptic area, astrocytic glucosensing may contribute to the sleep‐inducing effect of food intake (Scharbarg, Daenens, Lemaitre, Geoffroy, Guille‐Collignon, Gallopin, & Rancillac, 2016). In this context, tanycytes represent a further mechanism and cellular pathway for glucosensing in the brain. While some tanycytes may well use the pancreatic β cell mechanism of glucosensing, well documented for the glucose‐excited neurons, they also introduce a fundamentally new mechanism of central glucosensing dependent on the sweet taste receptor. Tanycytes add distinctive dimension to this menagerie of glucosensing cells as they contact the CSF and may be specifically tuned to detection of glucose within the CSF rather than the brain parenchyma (Bolborea and Dale, 2013; Frayling et al., 2011). It is important to establish whether this information from tanycytes is relayed to the neuronal circuits of the arcuate and ventromedial hypothalamic nucleus where glucosensitive neurons are located and to understand how these two pathways integrate to control feeding behavior and energy homeostasis.

## Supporting information

Supporting Information Movie 1

Supporting Information Movie 2

Supporting Information Movie 3
